# The impact of a disease management program (COACH) on the attainment of better cardiovascular risk control in dyslipidaemic patients at primary care centres (The DISSEMINATE Study): a randomised controlled trial

**DOI:** 10.1186/1471-2296-13-97

**Published:** 2012-10-10

**Authors:** Francis Jude Selvaraj, Mafauzy Mohamed, Khairani Omar, Sudha Nanthan, Zainab Kusiar, Selvaraj Y Subramaniam, Norsiah Ali, Kamalakaran Karanakaran, Fauziah Ahmad, Wilson H H Low

**Affiliations:** 1 Eli Lilly (M) Sdn Bhd, E1-2 Puncak Arabella, Tanah Rata, Cameron Highlands, Petaling Jaya, 39000, Malaysia; 2 Hospital University Science Malaysia, Kota Bharu, Kelantan, Malaysia; 3 National University Medical Center, Kuala Lumpur, Malaysia; 4 Polyclinic & Surgery Ayer Molek, Melaka, Malaysia; 5 Nilai Health Clinic, Nilai, Negeri Sembilan, Malaysia; 6 Awana Kijal Clinic, Kuala Terengganu, Terengganu, Malaysia; 7 Tampin Health Clinic, Tampin, Negeri Sembilan, Malaysia; 8 Kapar Clinic, Kapar, Selangor, Malaysia; 9 Bagan Serai Health Clinic, Bagan Serai, Perak, Malaysia; 10 Azmi Burhani Consulting, Petaling Jaya, Malaysia

**Keywords:** Dyslipidaemia, Disease management, Patient support, Cardiovascular risk control

## Abstract

**Background:**

To evaluate the efficacy of Counselling and Advisory Care for Health (COACH) programme in managing dyslipidaemia among primary care practices in Malaysia. This open-label, parallel, randomised controlled trial compared the COACH programme delivered by primary care physicians alone (PCP arm) and primary care physicians assisted by nurse educators (PCP-NE arm).

**Methods:**

This was a multi-centre, open label, randomised trial of a disease management programme (COACH) among dyslipidaemic patients in 21 Malaysia primary care practices. The participating centres enrolled 297 treatment naïve subjects who had the primary diagnosis of dyslipidaemia; 149 were randomised to the COACH programme delivered by primary care physicians assisted by nurse educators (PCP-NE) and 148 to care provided by primary care physicians (PCP) alone. The primary efficacy endpoint was the mean percentage change from baseline LDL-C at week 24 between the 2 study arms. Secondary endpoints included mean percentage change from baseline of lipid profile (TC, LDL-C, HDL-C, TG, TC: HDL ratio), Framingham Cardiovascular Health Risk Score and absolute risk change from baseline in blood pressure parameters at week 24. The study also assessed the sustainability of programme efficacy at week 36.

**Results:**

Both study arms demonstrated improvement in LDL-C from baseline. The least squares (LS) mean change from baseline LDL-C were −30.09% and −27.54% for PCP-NE and PCP respectively. The difference in mean change between groups was 2.55% (p=0.288), with a greater change seen in the PCP-NE arm. Similar observations were made between the study groups in relation to total cholesterol change at week 24. Significant difference in percentage change from baseline of HDL-C were observed between the PCP-NE and PCP groups, 3.01%, 95% CI 0.12-5.90, p=0.041, at week 24. There was no significant difference in lipid outcomes between 2 study groups at week 36 (12 weeks after the programme had ended).

**Conclusion:**

Patients who received coaching and advice from primary care physicians (with or without the assistance by nurse educators) showed improvement in LDL-cholesterol. Disease management services delivered by PCP-NE demonstrated a trend towards add-on improvements in cholesterol control compared to care delivered by physicians alone; however, the improvements were not maintained when the services were withdrawn.

**Trial registration:**

National Medical Research Registration (NMRR) Number: NMRR-08-287-1442

Trial Registration Number (ClinicalTrials.gov Identifier): NCT00708370

## Background

Chronic diseases pose a significant disease burden causing 60% of all deaths worldwide [1]. Of these, 50% are attributed to cardiovascular diseases [1]. Low-and middle-income countries are the biggest contributors to the increase in cardiovascular disease burden [2]. Although it varies among countries, the factors contributing to the escalating prevalence of chronic diseases are an ageing population, tobacco use, unhealthy diet practices and physical inactivity, urbanisation and global marketing [3], where half of these risk factors are modifiable through behaviour modification.

Primary healthcare plays a pivotal role in gearing patients towards positive behaviour management [4]. This can be achieved through the use of a chronic disease management (CDM) model, which emphasises the integration of several elements including multidisciplinary care delivery, patient education and provider decision support, self-management and patient empowerment support, clinical information technology, social support and quality incentives within the primary health care system [5]. Although studies have shown that chronic disease management are associated with marked improvements in many clinical outcomes associated with cardiovascular diseases [6-9]; many developing countries have yet to integrate CDM into their primary healthcare systems due to limited resources and systems orientated towards acute symptomatic care [4]. In addition, there is paucity of literature that addresses the sustainability of chronic disease management programmes in developing countries, in terms of its efficacy and cost.

Malaysia is not immune to the rising tide of chronic diseases, where cardiovascular diseases account for more than 25% of all-cause mortality [10] and prevalence of cardiovascular risk factors such as hypertension, diabetes mellitus and dyslipidaemia has reached epidemic proportions [11]. As a developing country, Malaysia is faced with the expected challenges of implementing chronic disease management in a resource-limited environment [12]. Although there have been several disease management programmes implemented in Malaysia, evidence on their cost-effectiveness, applicability and sustainability is lacking [13]. Despite the often-repeated recommendations to incorporate multidisciplinary healthcare teams in chronic disease care, access to allied health services is usually limited in a developing country like Malaysia. In addition, evidence to support the use of nurse-assisted dyslipidaemia management has been conflicting [14,15].

In view of the current lack of evidence on CDM efficacy in a developing country such as Malaysia, we designed a randomised controlled trial to assess the impact of a chronic disease management programme, COACH (Counselling and Advisory Care for Health) in managing dyslipidaemia. The COACH programme used in this study was intended to be modelled after the original COACH study [16,17]; however it was adjusted to the local situation and limitations. The primary objective of the DISSEMINATE study was to evaluate the efficacy of the COACH programme led by primary care physicians with assistance from nurse educators (PCP-NE) compared to that led by the primary care physicians alone (PCP) in improving serum low density level-cholesterol (LDL-C) in dyslipidaemic subjects over a period of 24 weeks. Secondary objectives included the impact of the COACH programme in the two arms in improving (i) patients’ lipid profiles i.e. High Density Lipoprotein-Cholesterol (HDL-C), LDL-C, Total Cholesterol (TC), Triglycerides (TG) and TC: HDL ratio, (ii) blood pressure (systolic and diastolic), (iii) Framingham cardiovascular risk, (iv) lifestyle modification (smoking behaviour, diet, alcohol consumption, physical activity), (v) programme satisfaction using a visual analogue scale (VAS) and (vi) statin compliance. All outcomes were assessed at 24 and 36 weeks of study duration. The outcomes assessment at 36 weeks was designed to evaluate the sustainability of programme effectiveness after the programme ended at week 24.

## Methods

### Trial design

This was a multi-centre, open-label, parallel, randomised trial of a disease management programme (COACH) led by primary care physicians for dyslipidaemic patients. Comparison was made between primary care physicians who managed patients alone (PCP Group-Control arm) versus those who were assisted by nurse educators (PCP-NE Group-Intervention arm). Patients were allocated on a 1:1 ratio. The study was approved by Malaysian Medical Research Ethics Committee (MREC) (National Medical Research Registration (NMRR) Number: NMRR-08-287-1442) and was conducted in compliance with the Declaration of Helsinki and International Conference on Harmonization (ICH) Good Clinical Practice (GCP) guidelines, as well as local regulatory requirements. No major changes were made to the trial methodology after study initiation.

### Site selection

Sites were selected based on their ability to recruit patients and to commit to the project. In order to achieve the recruitment rate and to provide a fair representation of the patient population in Peninsula Malaysia, licensed primary healthcare centres around the Klang Valley and other suburban towns were invited. Other recruitment factors were availability of infrastructure such as internet line or fax machine, the investigator’s understanding of GCP, the availability of a site coordinator and previous clinical trial experience. Thirty five sites were approached and 21 sites actively participated in the study.

### Subject recruitment

Patients who enrolled were newly diagnosed with dyslipidaemia. Subjects could be either male or female age 18 years or older. Besides having given informed consent, subjects must have been contactable via telephone, live or through short messaging services (SMS). Communication with patients through these methods was a key element of the COACH programme. Exclusion criteria applied included participation in any other clinical research studies in the preceding 6 months, history of mental illness, hypothyroidism, or presence of any other condition, which the investigators judged could increase risk to the subject and could interfere with the conduct of the study or interpretation of the data.

Eligibility was defined by dyslipidaemia divided according to 3 cardiovascular risk groups. Group I subjects were dyslipidaemic with an LDL-C level within 160 to 250mg/dL without concomitant cardiovascular risk factors. Group II subjects had at least one additional cardiovascular risk factor, excluding coronary heart disease (CHD) and diabetes mellitus (DM) with serum LDL-C level between 130 to 250mg/dL; Group III subjects consisted of those with serum LDL-C level between 100 to 250mg/dL with pre-existing CHD or CHD risk equivalent, such as DM or other atherosclerotic diseases. The study subjects were required to be lipid drug naïve and eligible for statin therapy prior to study enrolment. Random numbers were generated by computer and printed in sealed envelopes. The envelopes were subsequently allocated to each new patient enrolled into the programme. A person independent of the study was tasked to assign a randomisation number to a subject with known study identification number. The allocation of the individual study group was then made known when the envelopes were unveiled. Since COACH was administered by the nurse educators independent of the primary care doctor, knowledge of treatment allocation only became evident to the doctor when the subject returned for his/her third scheduled visit (week 12). The study duration was 36 weeks.

### Study procedure

All subjects received standard care and advice from their primary care physicians as per the National Cholesterol Education Program (NCEP) Expert Panel on Detection, Evaluation, and Treatment of High Blood Cholesterol in Adults (Adult Treatment Panel [ATP] III) guidelines [18]. As part of the standard care, all subjects received a COACH health booklet which was completed by the investigator during clinic visits. Subjects were expected to attend clinic visits at week 12, 24 and 36 after baseline randomisation. Statin dose was titrated according to cardiovascular risk group as recommended by NCEP ATP III Guidelines. Demographic data, primary diagnosis, medical history, family history, smoking status, alcohol, diet, physical activity, drug allergies and all other concomitant medications were captured in the study case report form (CRF). Blood pressure reading, pulse rate, general physical examination, 12-lead ECG, and blood sampling were also conducted during study visits. All blood samples were sent to a central laboratory for analysis. Telephone follow-up were made by site coordinators at week 6 and 18 to ensure all patients completed the health booklet correctly and complied with statin treatment. Subject compliance to prescribed statin was assessed by the primary care doctor and percentage compliance was noted in the CRF.

### Study intervention

In addition to the standard care, subjects randomised to the PCP-NE COACH Programme received bi-weekly telephone follow-up by trained nurse educators for 24 weeks. The main purpose of the telephone call was to provide patient’s self-management support and patient empowerment and the discussion was guided by the health education booklet. During the telephone follow-up, the nurse educators provided reinforcement of the health education information and reminded patients to adhere to counselling advice and prescribed medications, as well as to discuss any problems of adherence encountered with their physicians. Each telephone call lasted an average of 15 min. Subjects also received phone calls and SMS to remind them about forthcoming follow-up visits. The study intervention was designed to support the doctor-patient relationship and reinforce the prescribed care plan.

### Study control

All subjects randomised to PCP COACH Programme only received care from the site investigators as per normal practice. There was no additional telephone follow-up and reinforcement by nurse educators. At the end of 24 weeks, the study period ended. However, all subjects were required to attend follow-up visit at week 36, an extension phase, during which both arms received standard care only. This was to evaluate whether there would be sustained lipid control without reinforcement.

### Outcome measures

The primary efficacy endpoint was the mean percentage change from baseline LDL-C at week 24 between the two treatment arms. Other efficacy endpoints included mean percentage change from baseline of lipid profile (TC, LDL-C, HDL-C, TG, TC: HDL ratio), Framingham Cardiovascular Heart Risk Score, and absolute risk change from baseline in blood pressure parameters (systolic and diastolic) at week 24 and week 36. Lifestyle change assessment of smoking behaviour, food consumption, physical activity, alcohol consumption, and medication compliance were also evaluated at the end of the study.

### Statistical analysis

The sample size was calculated to detect a clinically meaningful difference of 10% in percentage change from baseline in LDL-C levels with a standard deviation of 22.5%, a 2-sided, 5% significance level and 85% power. During the implementation of the study, it was found that there was potential treatment contamination of the standard care arm where, behaviour modification occurred due to subjects from both arms interacting with each other. To overcome this, the sample size was adjusted to account 20% of treatment contamination (changing the effect size to 0.08%) and 10% attrition rate, giving the study a total of 320 subjects.

The analysis was conducted using intention-to-treat (ITT) analysis, which was defined as all lipid drug naïve subjects who were newly diagnosed with dyslipidaemia, eligible for statin therapy and were randomised with at least 1 post-baseline response (efficacy or outcomes research endpoints). Last observation carried forward was used for missing data in the ITT analysis.

Statistical analysis was based on the mean percentage change from baseline using a restricted maximum likelihood (REML) model with repeated measures approach (mixed model repeated measures [MMRM]). Significance tests were based on the least squares (LS) mean difference between treatment group to compare treatment contrast for week 24 and week 36. The analyses conducted at 24 weeks evaluated the effectiveness of the programme while analyses at 36 weeks assessed the medium-term effectiveness of care by physicians assisted by nurse educator (PCP-NE) compared with primary care physician care alone (PCP).

## Results

**Figure 1:**
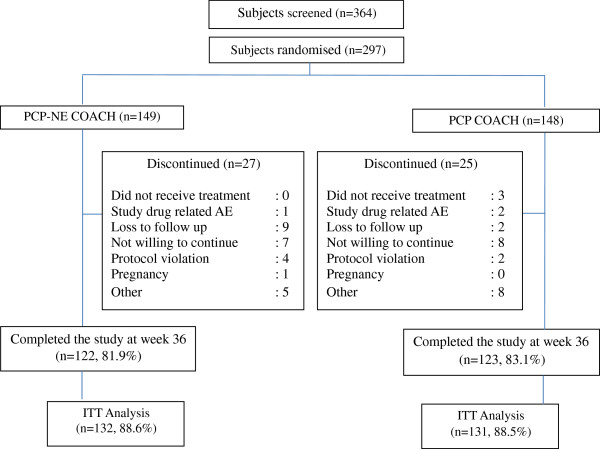
Study trial profile.

In order to achieve a sample size of 320 subjects, 364 potential subjects were screened however, only 297 subjects met the eligible criteria and were randomised. The period of patient recruitment was from September 2008 to July 2009. All patients were followed up for 36 weeks and the study was concluded in May 2010. One hundred and forty nine subjects were randomised to the PCP-NE COACH programme (Intervention) and 148 subjects to PCP COACH group (control). Of these, 122 (81.9%) from the intervention group and 123 (83.1%) from the standard care group completed the 36 weeks study. Figure 1 illustrates the trial profile for the DISSEMINATE study.

There were no significant differences in subjects’ baseline demographic characteristics and cardiovascular risk factors (Table 1). All subjects had a primary diagnosis of dyslipidaemia with a mean duration of 0.6 years from disease onset in both groups. All subjects were prescribed with HMG-CoA reductase inhibitors (statins). The most common statins prescribed in each group were lovastatin, simvastatin and atorvastatin. The median duration of statin treatment was 246 days. Approximately 99% and 95% of the patients in the intervention and control arm respectively reported compliance to statin treatment.

**Table 1:** Baseline subjects demographics & characteristics

	**Intervention Arm (n=149)**	**Control Arm (n=145)**
Number of subjects; n (%)		
Male	86 (57.7%)	83 (57.2%)
Female	63 (42.3%)	62 (42.8%)
Age, years; Mean (SD)	49.4 (11.1)	48.8 (9.9)
Race; Asian	149 (100.0)	145 (100.0)
Weight, kg; Mean(SD)	72.7 (15.2)	70.2 (15.1)
Height, cm; Mean (SD)	160.2 (8.2)	159.6 (9.3)
Body mass index, kg/m^2^; Mean (SD)	28.2 (4.9)	27.4 (4.9)
Mean duration of dyslipidaemia since onset (years)	0.6	0.6
Cardiovascular risk group ^a^; n (%)		
Group I	14 (9.4)	24 (16.6)
Group II	87 (58.4)	74 (51.0)
Group III	48 (32.2)	47 (32.4)
Age risk; n (%)		
Male aged >45; Female aged >55	72 (48)	60 (41)
Male aged ≤45; Female aged ≤55	77 (52)	85 (59)
Family history ^b^; n (%)		
Yes	29 (19)	32 (22)
No	120 (81)	113 (78)
Blood pressure ≥ 140/90 mm Hg or antihypertensive medication at baseline; n (%)		
Yes	89 (60)	88 (61)
No	60 (40)	57 (39)
Diabetes Mellitus; n (%)		
Yes	47 (32)	47 (32)
No	102 (68)	98 (68)
CHD or CHD risk equivalent ^a^; n (%)		
Yes	1 (1)	1 (1)
No	148 (99)	144 (99)
Smoking Status		
Never smoked	101 (68)	97 (67)
Ex-smoker	13 (9)	19 (13)
Current smoker	35 (23)	29 (20)

*COACH* = Counselling and Advisory Care for Health; *SD* = standard deviation; *CHD* = coronary heart disease; *PCP* = Primary Care Physicians; *PCP-NE* = Primary Care Physicians assisted by Nurse Educators.

^a^ Group I: subjects with dyslipidaemia with no other cardiovascular risk factors; Group II: subjects with dyslipidaemia with at least 1 additional cardiovascular risk factor, excluding CHD and DM; Group III: subjects with dyslipidaemia with CHD or CHD risk equivalent.

^b^ A history of premature coronary heart disease in a first-degree relative (parent or sibling). If the afflicted family member was male, premature coronary heart disease would have occurred before age 55, and if female, before age 65.

At the end of the study, the ITT set consisted of data from 132 and 131 subjects, from the intervention and control arms respectively. At week 24, there was a trend towards greater improvement in the intervention group. The least squares (LS) mean changes from baseline LDL-C were −30.09% and −27.54% for the intervention group and control groups, respectively. The difference of mean change in the intervention group was 2.55% lower than the control group, however this was not significant (p=0.288). At week 36, the LS mean of LDL-C between the 2 study arms was comparable at −25.88 and −26.86, for the intervention and control groups respectively. On the other hand, there was a statistically significant difference when comparing the LS mean in the intervention arm at week 24 and week 36 (p=0.016). This was due to withdrawal of the COACH programme which resulted in a diminution of the earlier LDL-C improvements seen in the intervention arm.

Similar outcomes were observed with total cholesterol (TC) level. The trend towards improved TC seen in the intervention group compared with the control arm at 24 weeks did not sustain at 36 weeks. No significant difference was detected in triglyceride (TG) levels at week 24 and 36 between the 2 study arms. Table 2 and 3 illustrates the percentage change from baseline in LDL-C and TC between the intervention and control group.

**Table 2:** Percent change from baseline in LDL-C, TC and HDL-C between intervention and control groups

**Time point**	**Outcomes**	**LS Mean (SE)**		**Difference in LS Means (%)**		
		**PCP-NE (n=132)**	**PCP (n=131)**	**PCP-NE versus PCP**	**95% Cl (%)**	**p-value**
Week 24	LDL-C	−30.09 (2.03)	−27.54 (2.03)	−2.55	−7.26, 2.16	0.288
	TC	−22.97 (1.55)	−20.80 (1.54)	−2.17	−5.72, 1.38	0.229
	HDL-C	−2.60 (1.30)	−5.61 (1.30)	3.01	0.12, 5.90	0.041
Week 36	LDL-C	−25.88 (2.03)	−26.86 (2.03)	0.98	−3.68, 5.65	0.679
	TC	−19.94 (1.53)	−19.97 (1.52)	0.04	−3.43, 3.51	0.984
	HDL-C	−2.97 (1.42)	−5.44 (1.41)	2.48	−0.79, 5.74	0.136

*LS* = Least Squares; *SE* = Standard Error; 95%; *CI* = 95% Confidence Interval; *LDL-C* = Low Density Lipoprotein Cholesterol; *TC* = Total Cholesterol; *HDL-C* = High Density Lipoprotein Cholesterol; *PCP* = Primary Care Physicians; *PCP-NE* = Primary Care Physicians assisted by Nurse Educators.

**Table 3:** Percent change from baseline in LDL-C, TC and HDL-C between week 24 and 36

**Study Arm**	**Outcomes**	**LS Mean (SE)**		**p-value**
		**Week 24**	**Week 36**	
PCP-NE (n=132)	LDL-C	−30.09 (2.03)	−25.88 (2.03)	0.016
	TC	−22.97 (1.55)	−19.94 (1.53)	0.018
	HDL-C	−2.60 (1.30)	−2.97 (1.42)	0.768
PCP (n=131)	LDL-C	−27.54 (2.03)	−26.86 (2.03)	0.695
	TC	−20.80 (1.54)	−19.97 (1.52)	0.515
	HDL-C	−5.61 (1.30)	−5.44 (1.41)	0.896

*LS* = Least Squares; *SE* = Standard Error; 95% *CI* = 95% Confidence Interval; *LDL-C* = Low Density Lipoprotein Cholesterol; *TC* = Total Cholesterol; *HDL-C* = High Density Lipoprotein Cholesterol; *PCP* = Primary Care Physicians; *PCP-NE* = Primary Care Physicians assisted by Nurse Educators.

In contrast, a significant difference was detected in high density lipoprotein-cholesterol (HDL-C) at week 24 between the intervention and control group with a greater decrease in HDL. As illustrated in Table 2, the difference in LS means of HDL-C between the intervention and control group was 3.01 (95% CI 0.12 – 5.90), p=0.041 at week 24. However, this effect did not persist beyond the duration of the intervention (p=0.136) after week 24 (Table 3). These results at and after week 24 were also reflected similarly in the total cholesterol (TC): high-density lipoprotein cholesterol (HDL-C) ratio.

There were no significant differences between both study arms in relation to statin treatment compliance, systolic and diastolic blood pressure and Framingham Coronary Heart Disease risk scores at week 24. In terms of lifestyle modification (i.e. cigarettes smoking, dietary changes, and physical activity), no difference was observed between the intervention and control group. In general, more than 80% of the subjects were satisfied with the health booklet provided. Approximately 90% of subjects from the intervention arm also expressed satisfaction with the programme in helping them achieve health care goals through telephone follow-up by nurse educators.

## Discussion

The COACH programme in this study utilised patient education and empowerment as well as decision support as interventional strategies to improve cholesterol control. Both study arms had shown improved LDL-C and TC level throughout the study period. The results were a trend towards add-on improvements in both LDL-C and TC levels when patients were co-managed by nurse educators, even though this was not statistically significant.

The lack of statistical significance was most likely attributed to dilution of treatment effects with the use of similar patient education methods in the control group as well as in the intervention group, specifically, using the COACH health booklet. The COACH programme applied in our study delivered more comprehensive care than usual compared to the typical Malaysian primary care settings, which usually have little time or resource to provide comprehensive disease management care as part of daily practices [19]. As a result, in this study, patients from both study arms would have benefited from increased knowledge of their health conditions.

Although the COACH programme used in this study was similar to other studies published by Vale et al. [16] and Allen et al. [17], there are significant differences in terms of study methodology. In the study conducted Vale et al. [16], patients were continuously coached based on previous assessment visit and progress monitored via negotiated action plan. In addition, the Vale’s study focused on hospital based disease management programme. On the other hand, the COACH programme by Allen et al. [17] utilised the model of community-based participatory research methodology to design the disease management programme, which consisted of enhanced usual care with or without intensive disease management by nurse practitioner/community health workers. Both studies had adopted many essential elements from the Chronic Care Model [3], which had been proven to improve chronic disease outcomes. In contrast to this study, the authors had only adapted the previous COACH studies as a patient support programme rather than a disease management programme due to severe resource limitation i.e. lack of trained nursing support resulting in resistance from local doctors to adopt a shared-care model on patient disease management counselling. As a result, the nurse educators in this study had no access to patient’s medical records and health education was reinforced using the health education booklet only.

Evidence for the success of disease management programmes involving additional support from nurses or other disciplines is mixed. Our findings are comparable with some studies [14,20] where reduction in blood cholesterol was similarly equivocal due to the Hawthorne effect among patients and healthcare providers, a change in people’s behaviour when being observed. In addition, the study may have heightened the awareness of disease management practices among family physicians involved. However, studies published by Vale et al. [16] and Allen et al. [17] that utilised a similar programme, demonstrated significant improvement in LDL-C and TC change from baseline when patients with coronary heart diseases underwent the programme. As opposed to our study, the positive outcomes demonstrated in both the studies may be due to: (1) the population enrolled i.e. the study recruited CHD patients experiencing acute coronary syndrome who were more motivated to change; (2) difference in setting i.e. developed versus developing country; (3) difference in prescription behaviour i.e. higher proportion of patients prescribed with statin and with higher doses of atorvastatin in the Vale et al. study (not commonly prescribed among the doctors in the DISSEMINATE study). Besides the 2 studies discussed above, there are also other studies that have had positive results with multi-disciplinary support [21-24].

The DISSEMINATE study had also revealed interesting findings with regards to HDL-C decrease over the 6 months duration. This finding was inconsistent with many clinical studies that reported mild improvement of HDL-C with both drug treatment and disease management care [25,26]. Several theories are hypothesised for this outcome. First of all, there is some evidence that a low fat diet reduces not just LDL-C but also HDL-C [27-29]. Secondly, the COACH programme did not provide detailed nutritional education with information on how to reduce high dietary fat intake while substituting dietary fat with polyunsaturated fatty acids (PUFA) such as olive oil. It is possible that both of these factors could have resulted in the unexpected reduction in HDL-C levels detected in our study.

Several limitations to our study are identified. In an effort to standardise patient care between different sites, a health booklet was distributed to all subjects. As discussed above, this had diluted the difference in interventional effects between control group and intervention group. Also, the findings in the control arm would not be generalise to the current primary care settings as it had deviated from the normal local practice; however, the outcomes from this study has demonstrated that current chronic disease care in Malaysia primary care practices are suboptimal and some improvements in patient’s disease outcome can be achieved by simply spending extra time to educate the patients on chronic disease self-management and treatment compliance. And furthermore, our study has also shown that the task of personalised patient education does not have to be purely the domain of the physicians but can also be provided by trained nurses. The study might have elicited a more significant finding with addition of a third study arm assessing the lipid outcomes among patients who received “actual” standard care delivery by local physicians. Though the primary outcome of the study was not achieved, an additional post-hoc analysis may be able to determine the proportion of uncontrolled dyslipidaemic patients who achieved cholesterol target at the end of the study follow-up. In the current analysis, the proportion of patients who achieved target cholesterol level is not known. A higher than expected attrition rate (initial sample size calculation only accounted for 10% loss to follow-up) could have also affected the final results of the study.

Although our study showed that complementary nurse support services in a disease management programme had a positive trending impact on LDL-C and TC level compared to physician management alone, the effect was not sustainable after the intervention was withdrawn at week 24. Continuous therapeutic behavioural change seems to be mandated to ensure long-term sustainable lipid control.

## Conclusion

This study was designed as a trial in a “real-world” setting and thus faced with its share of expected challenges. The limitations of our study are acknowledged, and some of the lack of effect may be attributable to similarities between the management of patients in the intervention and control group. Further study would be warranted to build upon the results of this study while addressing some of the limitations. Despite the lack of statistical significance, the trend towards improved dyslipidaemia can be considered a success at some level. As there is a paucity of published evidence on disease management programmes in this region, the data provided by this study may be considered an indicator about the potential role of disease management programmes in developing Asian countries.

## Abbreviations

AE: Adverse events; ATP: Adult Treatment Panel; BMI: Body mass index; BP: Blood pressure; CDM: Chronic Disease Management; CHD: Coronary heart disease; CI: Confidence interval; Cm: Centimeter; COACH: Counselling and Advisory Care for Health; CORFIS: Community based cardiovascular risk factor intervention strategy; CRF: Case report form; DISSEMINATE: Disease management programme on the attainment of better cardiovascular risk control; DM: Diabetes mellitus; ECG: Electrocardiogram; ESRD: End stage renal disease; FAS: Full analysis set; GCP: Good Clinical Practice; GP: General practitioner; HDL-C: High density lipoprotein cholesterol; HMG-CoA: 3-Hydroxy-3-Methyl-Glutaryl-CoA; ICH: International Conference on Harmonization; Kg: Kilogram; LDL-C: Low density lipoprotein cholesterol; LS: Least squares; mg/dL: Milligram per deciliter; mmol/L: Millimol per liter; mmHg: Millimeter mercury; MMRM: Mixed model repeated measures; MREC: Medical Research Ethical Committee; NCEP: National Cholesterol Education Program; NIH: National Institute of Health; PUFA: Polyunsaturated fatty-acid; REML: Restricted maximum likelihood model; SD: Standard deviation; SE: Standard error; SMS: Short messaging service; TC: Total cholesterol; TG: Triglycerides; VAS: Visual analogue scale; WHO: World Health Organization.

## Competing interests

The preparation of this manuscript was funded by a grant from Pfizer (Malaysia) Sdn Bhd. However, Pfizer has no influence over the drafting of the manuscript, nor the decision to publish. The responsibility for the contents of this manuscript rests entirely with the authors. The authors and investigators in this study have no association with the original COACH programme. All authors were independent investigators of the study with the exception of JFS who was previously an employee of Pfizer (M) Sdn Bhd.

## Authors’ contributions

All authors have contributed significantly to the success of this study. JFS was responsible for the conception and the design of the study as well as reviewed the manuscript. MM, KO, SN, ZK, SYS, NA, KK and FA demonstrated significant input and dedication during patient recruitment and data collection. WL was responsible for conducting the literature review and manuscript write up. All authors read and approved the final manuscript.

## Pre-publication history

The pre-publication history for this paper can be accessed here:

http://www.biomedcentral.com/1471-2296/13/97/prepub
